# Book Reviews

**Published:** 1893-09

**Authors:** 


### Book Reviews.

The Structures in the Mesosalpinx: Their Normal and Pathological Anatomy. By J. W. Ballantyne, M. D., F. R. C. P. E., F. R. S. E., Lecturer on Midwifery and Diseases of Women, School of Medicine, Edinburgh; Secretary to the Edinburgh Obstetrical Society; and formerly Senior Assistant to the Professor of Midwifery and Diseases of Women and Children in the University of Edinburgh; and J. D. Williams, M. D., B. Sc., Freeland Barbour Fellow (Univ. Edin., 1888-90). Edinburgh: Oliver & Boyd, Tweeddale Court. 1893.

This is a capital little brochure upon the normal and pathological anatomy of the Fallopian tubes, and the organ of Rosenmüller. The authors preface the descriptions proper with a few remarks upon the mesosalpinx. This is a double fold of peritoneum, bounded above by the Fallopian tube, internally by the lateral wall of the uterus, externally by the tubo-ovarian fimbria, and the ligamentum infundibulo-ovaricumi of Henle, and inferiorly by the ovary and the utero-ovarian ligament. Irregularly triangular in form, its base is directed toward the side wall of the pelvis, its apex toward the uterus. Its average breadth at its widest part (junction of outer and middle third) is four centimeters; its average length is eight centimeters. It is not identical with the broad ligament, but is the superior or middle fold of the three making up the broad ligament. Its synonyms are a la nespertilionis (of the older anatomists), and mesentery of the Fallopian tube (modern). The structures within the mesosalpinx are the Fallopian tube, the relics of the mesonephros (organ of Rosenmüller), scattered smooth muscular fibers, and blood-vessels, lymphatics, and nerves.

The authors base their deductions upon the results of examination of 110 pairs of broad ligament taken from females of varying ages (fetal life to seventy-six years of age). The specimens were first examined by the naked eye by transmitted light; they were then placed in a weak solution of acetic acid, and, after three or four hours, examined again in the same way; then the posterior layer of the ligament was carefully dissected off under water and the structures accurately followed out. Finally, the specimen was placed in a preservative fluid for future microscopical examination. The Fallopian tubes are first considered. The histology is carefully given, and the interdependence of normal and pathological anatomy emphasized. Equally clear and instructive are the pages dealing with hypertrophy of the Fallopian tubes, hydro-, hemato-, and pyosalpinx, certain malformations and displacements of the tubes, tubercular disease, and cancerous affection of the tubes.

In the same intelligent way the macro- and microscopic character of the organ of Rosenmüller, the homologues of the mesonephoric relics are discussed. Among the pathological conditions of this organ here described are cysts, cancer, varicosity of the veins, and phleboliths. A few remarks upon backward displacement and cellulitic thickening of the mesosalpinx form the conclusion of this valuable pamphlet. The reader will find in it much that does not appear in ordinary text-books of anatomy of even recent date. It is excellently printed, and deserves a wide circulation.

J. P.

---

THE STUDENTS' QUIZ SERIES. Genito-Urinary and Venereal Diseases. A Manual for Students and Practitioners. By CHAS. H. CHETWOOD, M. D., Visiting Surgeon, Demilt Dispensary, Department of Surgery and Genito-Urinary Diseases, New York. Series edited by Bern. B. Gallaudet, M. D., Demonstrator of Anatomy, College of Physicians and Surgeons, New York; Visiting Surgeon, Bellevue Hospital, New York. Philadelphia: Lea Brothers & Co.

This work is one of the best of the Quiz compends, and aims to present the various subjects by questions which would be suggested to the student and practitioner, and to form the answers in a conversational and descriptive manner, avoiding terse summaries. The author draws from a large clinical experience in this department of medicine, and also from the standard authorities. He has succeeded in condensing, in a small compass, a mass of valuable data, which will aid the overworked student and also the busy practitioner. We regard such works as absolutely necessary, and conclude that this effort of the author is a very successful one.

THE STUDENTS' QUIZ SERIES. GYNECOLOGY. A Manual for Students and Practitioners. By G. W. BRATENAHL, M. D., Assistant in Gynecology, Vanderbilt Clinic, New York, and SINCLAIR TOUSEY, M. D., Assistant Surgeon, Out-Patient Department, Roosevelt Hospital, New York. Series edited by Bern. B. Gallaudet, M. D., Demonstrator Anatomy, College Physicians and Surgeons, etc. Philadelphia: Lea Brothers & Co.

The subjects treated in this compend are of great interest to the profession, and are here presented in a concise manner, the salient points alone being touched upon by the authors. The larger works on these subjects are in the library of every intelligent physician, and are often too exhaustive to meet the exigencies in his daily experience. It is objected that such works lead to a very superficial knowledge of the subjects, and, in a measure, this objection is well founded. But we think there is a field of usefulness filled by such compends, and, in this view of the case, conclude that the authors have done good service in presenting in a direct manner the main points of many interesting features of the department of medicine, to which the work is devoted.

---

THE STUDENTS' QUIZ SERIES. Diseases of the Eye, Ear, Throat, and Nose. A Manual for Students and Practitioners. By FRANK E. MILLER, M. D., Attending Physician, St. Joseph's Hospital; Throat Surgeon, Vanderbilt Clinic, New York. James P. McEvoy, M. D., Throat Surgeon, Bellevue Hospital, Out-patient Department, New York; and John E. Weeks, M. D., Surgeon New York Eye and Ear Infirmary; Lecturer on Ophthalmology and Otology, Bellevue Hospital Medical College, New York. Lea Brothers & Co.

A very useful compend, which contains much condensed information, useful both to the practitioner and the student. It would ordinarily be of more use to the practitioner than the student on account of the large amount of ground it covers. The anatomical and histological descriptions are good and complete. The definitions are clear and concise, and the treatment is very fully described. Taken as a whole, if it were not for the interpolations of the questions, it would answer very well for a text-book.

---

### BOOKS RECEIVED.

Report of the Commissioner of Education for the year 1889-1890. Volumes I. and II., containing Parts I., II., and III. Washington, D. C.: Government Printing Office. 1893.

Operation Blanks. Second edition. Prepared by W. W. Keen, M. D., Professor of the Principles of Surgery in the Jefferson Medical College, Philadelphia.

Transactions of the Medical Society of the State of New York for the year 1893. Published by the Society. 1893.

Weekly Abstract of Sanitary Reports. Issued by the Supervising Surgeon General, M. H. S., under the National Quarantine Act of April 29, 1878. Volume VII., Nos. 1 to 53. Washington: Government Printing Office. 1893.

Reactions. A Selection of Organic Chemical Preparations Important to Pharmacy in Regard to their Behavior to Commonly Used Reagents. By F. A. Fluckiger, Ph., M. D. Translated, revised, and enlarged by J. B. Nagelvoort, Analytical Chemist of the Parm. Chem. Laboratory of Parke, Davis & Co. Authorized English edition. Detroit, Mich.: George S. Davis. 1893.

Mineral Springs and Health Resorts of California, with a complete Chemical Analysis of every Important Mineral Water in the World. Illustrated. A Prize Essay. Annual Prize of the Medical Society of the State of California, awarded April 20, 1889. By Winslow Anderson, M. D., M. R. C. P., Lond., M. R. C. S., Eng., etc.; Joint Editor and Publisher of the *Pacific Medical Journal*, etc., etc. San Francisco: The Bancroft Co. 1892.

Bureau of Education Circular of Information No. 4. 1893. Abnormal Man. Being Essays on Education and Crime, and Related Subjects, with Digests of Literature and a Bibliography. By Arthur MacDonald, Specialist in the Bureau of Education. Washington: Government Printing Office. 1893.

